# The Bacterial Toxin CNF1 Protects Human Neuroblastoma SH-SY5Y Cells against 6-Hydroxydopamine-Induced Cell Damage: The Hypothesis of CNF1-Promoted Autophagy as an Antioxidant Strategy

**DOI:** 10.3390/ijms21093390

**Published:** 2020-05-11

**Authors:** Sara Travaglione, Stefano Loizzo, Rosa Vona, Giulia Ballan, Roberto Rivabene, Danila Giordani, Marco Guidotti, Maria Luisa Dupuis, Zaira Maroccia, Monica Baiula, Roberto Rimondini, Gabriele Campana, Carla Fiorentini

**Affiliations:** 1 Istituto Superiore di Sanità, 00161 Rome, Italy; stefano.loizzo@iss.it (S.L.); rosa.vona@iss.it (R.V.); giukkab@gmail.com (G.B.); roberto.rivabene@iss.it (R.Riv); danila.giordani.dg@gmail.com (D.G.); marco.guidotti@iss.it (M.G.); marialuisa.dupuis@iss.it (M.L.D.); zaira.maroccia@iss.it (Z.M.); carla.fiorentini@iss.it or; 2 University of Bologna, 40126 Bologna, Italy; monica.baiula@unibo.it (M.B.); roberto.rimondini@unibo.it (R.Rim); gabriele.campana@unibo.it (G.C.); 3 Association for Research on Integrative Oncology Therapies (ARTOI), 00165 Rome, Italy

**Keywords:** 6-hydroxydopamine, mitochondria, cytotoxic necrotizing factor type 1, oxidative stress, reactive oxygen species, macroautophagy, Rho GTP-binding proteins

## Abstract

Several chronic neuroinflammatory diseases, including Parkinson’s disease (PD), have the so-called ‘redox imbalance’ in common, a dynamic system modulated by various factors. Among them, alteration of the mitochondrial functionality can cause overproduction of reactive oxygen species (ROS) with the consequent induction of oxidative DNA damage and apoptosis. Considering the failure of clinical trials with drugs that eliminate ROS directly, research currently focuses on approaches that counteract redox imbalance, thus restoring normal physiology in a neuroinflammatory condition. Herein, we used SH-SY5Y cells treated with 6-hydroxydopamine (6-OHDA), a neurotoxin broadly employed to generate experimental models of PD. Cells were pre-treated with the Rho-modulating *Escherichia coli* cytotoxic necrotizing factor 1 (CNF1), before the addition of 6-OHDA. Then, cell viability, mitochondrial morphology and dynamics, redox profile as well as autophagic markers expression were assessed. We found that CNF1 preserves cell viability and counteracts oxidative stress induced by 6-OHDA. These effects are accompanied by modulation of the mitochondrial network and an increase in macroautophagic markers. Our results confirm the Rho GTPases as suitable pharmacological targets to counteract neuroinflammatory diseases and evidence the potentiality of CNF1, whose beneficial effects on pathological animal models have been already proven to act against oxidative stress through an autophagic strategy.

## 1. Introduction

Parkinson’s disease (PD) is the second most common neurodegenerative disease, disabling millions worldwide. Effective treatments to prevent and cure PD are still lacking [1]. Among the hallmarks in the pathogenesis of PD, a crucial role is played by neuroinflammation and redox imbalance, the latter being a dynamic system that may change via many factors [2], including mitochondria that are central to energy metabolism. Mitochondrial dysfunction is often accompanied by increased reactive oxygen species (ROS) production by the organelle itself [3], with the consequent formation of hydrogen peroxide. Together with other downstream products, ROS cause non-specific oxidative damage that has been linked with aging and also contributes to a wide variety of chronic diseases, including PD [4,5,6,7,8,9]. For this reason, over recent years drug research has focused on potential therapeutic tools able to restore physiologic conditions by counteracting ROS production and rescuing mitochondrial functionality. Treatments with general ROS scavengers, however, have multiple limitations that are probably implied in clinical failure [10,11,12]. On the other hand, anti-oxidants that directly target mitochondria though apparently are more efficacious in animal models are still unexplored in terms of their benefits in humans [13,14,15,16] and, furthermore, may also eliminate physiologically-relevant ROS [13,14,15,16] that act as key molecules in different signaling pathways [12]. To face this problem and then eliminate the production of pathological ROS, an effective strategy could be focused on mitochondrial dynamics, since the maintenance of a balanced fission/fusion cycle can prevent the accumulation of damaged and fragmented mitochondria and thus eliminate the production of pathological ROS [17].

In this context, the *Escherichia coli* protein toxin cytotoxic necrotizing factor 1 (CNF1) may represent a promising potential agent against several neurological diseases characterized by alterations in mitochondrial functionality and pathological ROS production [18,19,20,21,22]. CNF1 acts by deamidating Rho GTPases (Rho, Rac and Cdc42) on a specific glutamine residue inside the GTP-binding domain, thus blocking the molecules in their activated, GTP-bound state [23,24]. This persistent activation permits the toxin to modulate the actin cytoskeleton [25], a crucial cellular network that drives many aspects of cell behavior, including mitochondrial motility and functionality [26,27]. Previously, we showed that CNF1 can induce, in epithelial non-transformed cells, an increment of the mitochondrial electric transport chain activity with a consequent increase in ATP cellular production and content. Such an increase is accompanied by phosphorylation/inactivation of dynamin-related protein 1 (Drp1) that causes a profound modification of the mitochondrial architecture, consisting of the formation of a complex network of elongated mitochondria [18]. Interestingly, the CNF1 ability to trigger an increment of cell energy production has been confirmed in vivo in mouse models of PD [22], as well as of other neuroinflammatory diseases characterized by brain mitochondria impairment, such as Alzheimer’s disease [28], epilepsy [20] and Rett syndrome [29,30]. 

The purpose of this study was to evaluate the effects of CNF1 in SH-SY5Y cell cultures treated with 6-hydroxydopamine (6-OHDA), an oxidative stress-inducing agent broadly used to generate experimental models of PD [31]. It has been reported that the lack of expression of Drp1 in this in vitro model prevents mitochondrial fission [32] and that the pharmacological inhibition of Drp1 activity results in the abrogation of mitochondrial fission accompanied by modulation of the autophagic process [33], suggesting the involvement of autophagy in such a context. Since it is known that modulation of Rho GTPases’ activity can affect autophagy [34], we investigated whether a relationship would exist between the CNF1 neuroprotective action and cell autophagy in a stress-inducing in vitro system, with the final goal to suggest a novel and effective antioxidant strategy that involves the Rho GTPases against neuroinflammatory diseases.

## 2. Results

### 2.1. CNF1 Pre-Treatment Partially Rescues Cell Viability in SH-SY5Y Cells Exposed to the Neurotoxic 6-OHDA

**Figure 1:**
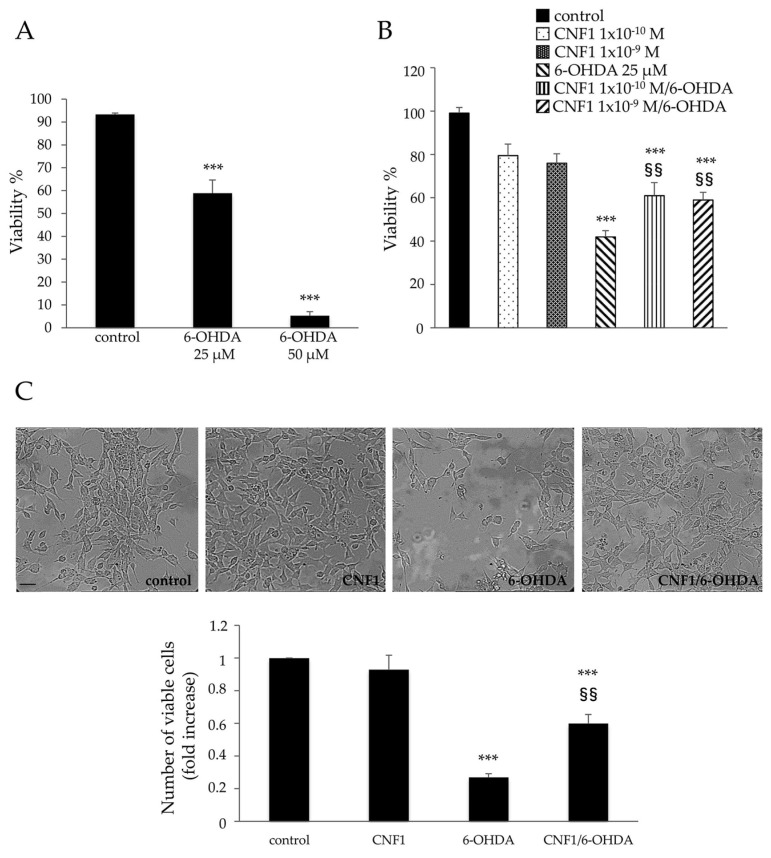
Cytotoxic necrotizing factor 1 (CNF1) partially rescues cell viability in SH-SY5Y cells exposed to neurotoxic 6-hydroxydopamine (6-OHDA). (**A**) Histogram showing cell viability quantified using MTS assay. At 25 μM, 6-OHDA significantly reduced cell viability (58.9 ± 5.4%), while 50 μM dosage resulted to be extremely toxic for cells. (**B**) Histogram showing the ability of CNF1 to partially counteract the cell toxicity induced by 6-OHDA. (**C**) Phase-contrast micrographs (upper panels) confirming the ability of CNF1 to counteract 6-OHDA-induced cell toxicity. Note that in cells pre-treated with CNF1, the cell density is very similar to that of control cells. Bottom panel: histogram showing viable cells (fold increase) quantified using the trypan blue exclusion method. Data on the graphs represent the mean ± SEM from at least three independent experiments. *** *p* < 0.001 compared with control; ^§§^ *p* < 0.01 compared with 6-OHDA. Bar = 10 μm.

We first performed a dose-response experiment by treating cells with two different doses of 6-OHDA (25 and 50 μM) and then quantified the cell viability after 24 h, using MTS assay (Figure 1A). Within 24 h of treatment with 6-OHDA at 25 μM, the majority of SH-SY5Y cells showed typical morphological changes, such as membrane blebbing and cell shrinkage (data not shown), without dramatically impairing cell viability (58.9 ± 5.4%), while the 50 μM dosage had extremely toxic effects for cells (Figure 1A). Therefore, a 25 μM dosage was chosen for inducing cell toxicity and evaluating the protective effects of CNF1.

Pre-incubation for 2 h with CNF1, at the concentrations of 1 × 10^−10^ M and 1 × 10^−9^ M, significantly prevented the cell toxicity induced by the subsequent addition of 6-OHDA for further 24 h. CNF1 alone apparently lowered viability in control cells, although the differences observed did not reach statistical significance. Finally, no statistical differences were observed between the two CNF1 doses (Figure 1B). Therefore, a 1 × 10^−10^ M concentration of CNF1 was selected for use in the next set of experiments. 

Phase-contrast micrographs (Figure 1C, upper line) confirm the ability of CNF1 to counteract 6-OHDA-induced cell toxicity. Indeed, in cells pre-treated with CNF1, cell density was very similar to that of control cells. This was quantified by counting viable cells using the trypan blue exclusion method (Figure 1C, histogram).

### 2.2. CNF1 Influences Mitochondrial Morphology and Dynamics in SH-SY5Y Cells

**Figure 2:**
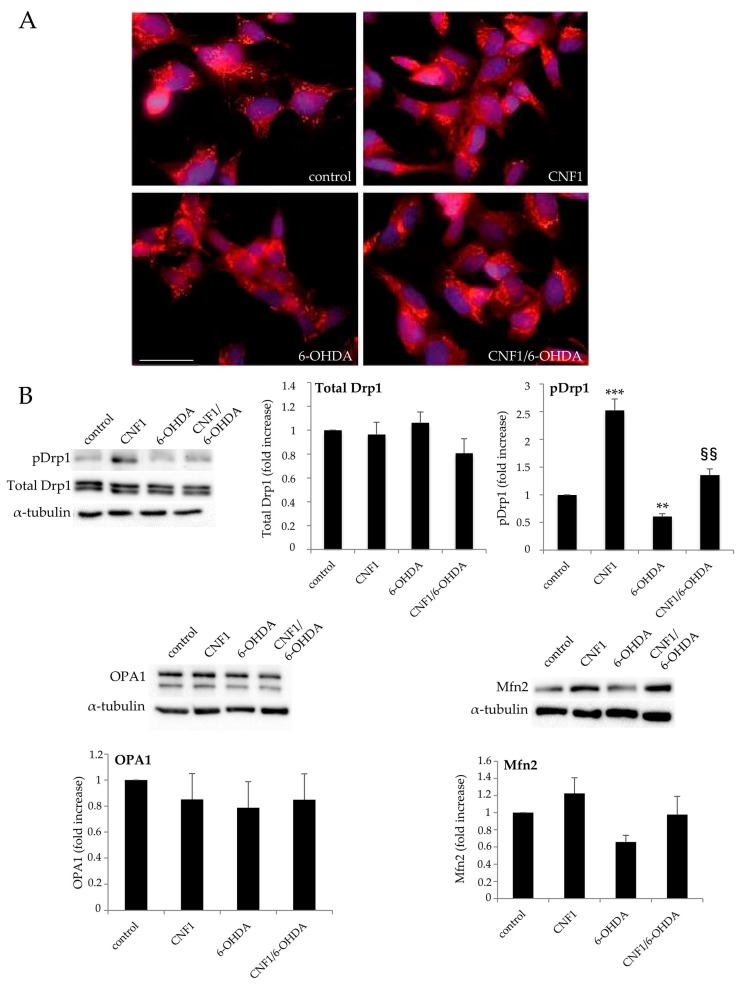
CNF1 influences mitochondrial morphology and dynamics in SH-SY5Y cells. (**A**) Fluorescence microscopy micrographs of SH-SY5Y cells in the different experimental conditions, co-stained with the mitochondrion-selective dye MitoTracker (red) and with the nuclear dye Hoechst 33258 (blue). Note that pre-treatment with CNF1 counteracts the neurotoxin-induced mitochondrial fragmentation, promoting an enrichment of the mitochondrial network, either in control or in 6-OHDA treated cells. Bar = 10 µm. (**B**) Western blot analysis in whole-cell lysates of proteins involved in mitochondrial fusion/fission proteins. The amounts of OPA1, Mfn2 and total Drp1 are normalized as a function of α-tubulin, while the amount of pDrp1 is normalized as a function of normalized total Drp1 (histograms). Note that CNF1 dramatically increases pDrp1 expression in control cells and counteracts the reduction of the phosphorylated protein in cells challenged with 6-OHDA. The graphs report the mean ± SEM from three different experiments. For each protein, results are expressed as fold increase relative to control (=1). *** *p* < 0.001 compared with control; ** *p* < 0.01 compared with control; ^§§^ *p* < 0.01 compared with 6-OHDA.

As reported in the Introduction, it is well established that mitochondrial dysfunctions are at the basis of different neurodegenerative pathologies, including PD [7,35]. Therefore, to assess the molecular mechanism underlining the neuroprotective action of CNF1, we investigated the toxin effect on mitochondrial dynamics in this in vitro model. Mitotracker staining (Figure 2A) showed that, while 6-OHDA-treated cells bear extremely fragmented and condensed mitochondria, pre-treatment with CNF1 counteracted the neurotoxin-induced mitochondrial fragmentation, promoting the enrichment of the mitochondrial network that consisted in elongated and interconnected organelles, either in control or in 6-OHDA treated cells. To unravel the mechanism involved in the CNF1-dependent mitochondria elongation, we evaluated the expression of proteins related to mitochondrial fission (Drp1) or fusion processes (OPA1, Mfn2). Western blot analyses (Figure 2B) evidenced that neither OPA1 nor total Drp1 expressions varied in the four experimental groups. By contrast, 6-OHDA treatment significantly reduced the expression of Drp1 protein phosphorylated in Ser^637^ (pDrp1). It is worth noting that protein kinase A (PKA)-dependent phosphorylation of Drp1 protein in Ser^637^ blocks Drp1 translocation on mitochondrial membranes, thus inhibiting the fission process [36,37]. As expected, CNF1 dramatically increased pDrp1 expression in control cells and, more importantly, counteracted the reduction of the phosphorylated protein in cells challenged with 6-OHDA. The expression of the mitochondrial fusion protein Mfn2 was affected similarly by CNF1, although the differences observed did not reach statistical significance.

### 2.3. CNF1 Counteracts 6-OHDA-Induced Oxidative Stress 

Cell death induced by 6-OHDA is not due to inhibition of mitochondrial energy supply, but probably caused by cell-free radicals production [38]. Therefore, we asked whether the beneficial preventive action of CNF1 on 6-OHDA-neurotoxicity could be due to an impact on ROS. To address this question, we studied the redox profile of SH-SY5Y cells in the four experimental groups analyzed.

**Figure 3:**
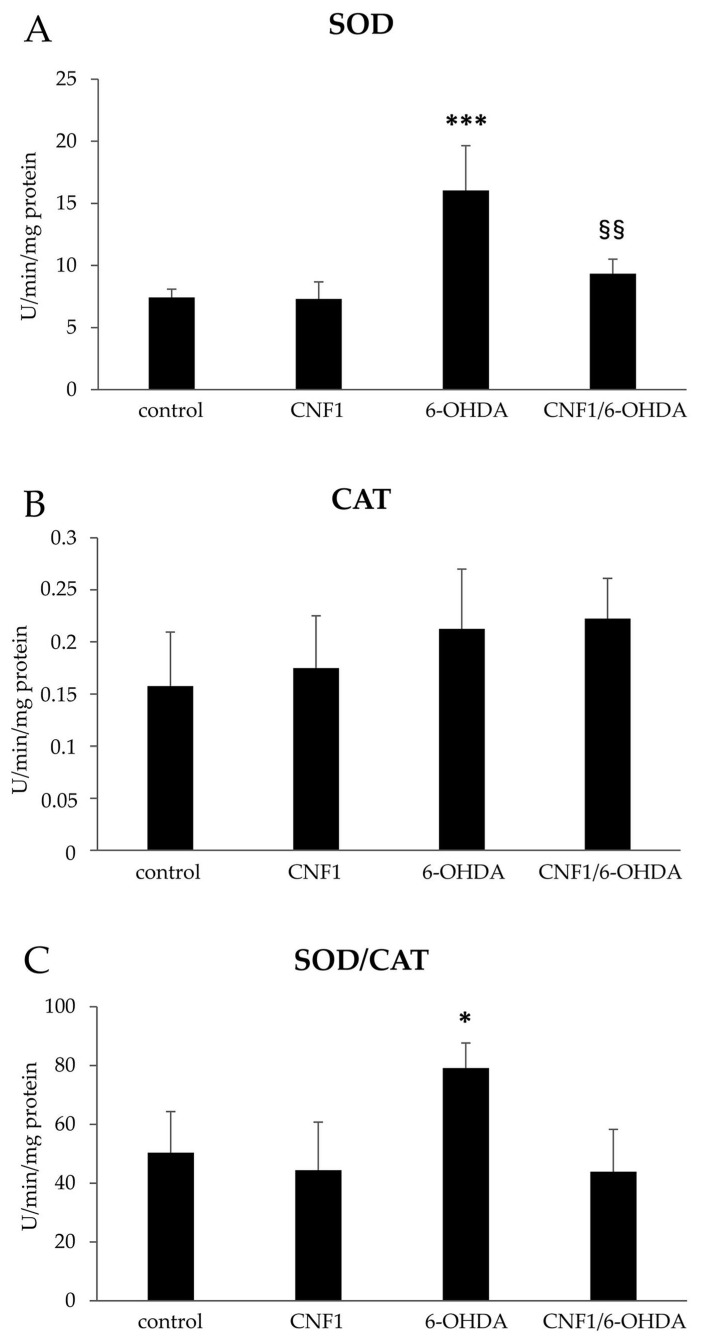
Evaluation of superoxide dismutase (SOD) and catalase (CAT) enzymatic activities in SH-SY5Y cells. (**A**) SOD activity; (**B**) CAT activity; (**C**) SOD/CAT ratio. The results are expressed as mean ± SEM from four independent experiments. *** *p* < 0.001 compared with control; ^§§^ *p* < 0.01 compared with 6-OHDA; * *p* < 0.05 compared with control.

Since among the endogenous antioxidant enzymes, superoxide dismutase (SOD) and catalase (CAT) constitute the first line of natural defense against pathological effects induced by an excess of free radicals, we measured their activity to evaluate the first impact of all treatments on the SH-SY5Y redox profile. As shown in Figure 3A, a consistent and significant increase in enzymatic activity of SOD in cells treated with 6-OHDA was observed. At the same time, cells treated with CNF1 alone did not modify basal SOD activity. Interestingly, cell pre-treatment with CNF1 was able to efficiently counteract the above-reported effects due to 6-OHDA exposure, with SOD activity values close to those reported for controls. As concerns the enzymatic activity of CAT, no significant differences were reported in the four experimental groups (Figure 3B). Changes in the equilibrium between the formation of H_2_O_2_ from superoxide dismutation and its decomposition by CAT can be expressed by the ratio R = SOD/CAT, which appeared to be significantly higher only in cells treated with 6-OHDA alone (Figure 3C). These findings suggest a SOD-dependent overload of H_2_O_2_ within 6-OHDA treated cells, which was not efficiently removed by CAT.

**Figure 4:**
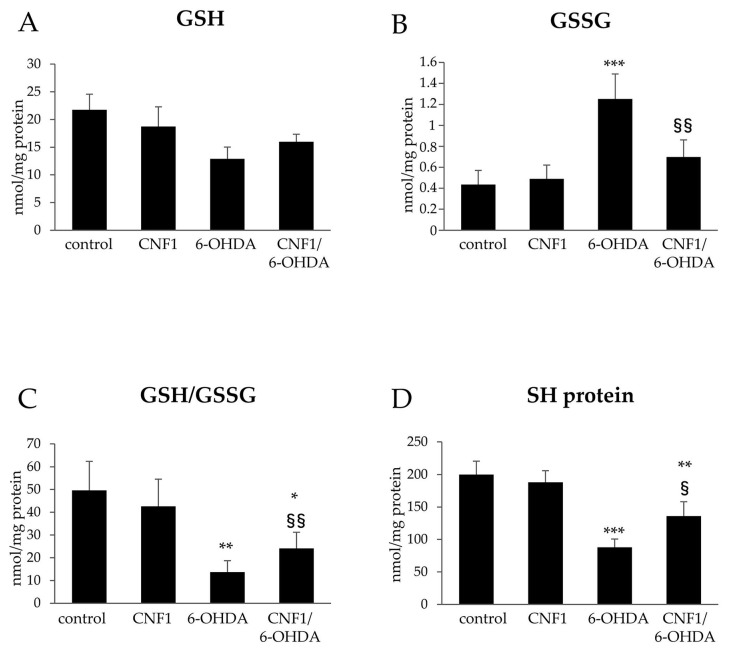
Evaluation of reduced glutathione (GSH), oxidized glutathione (GSSG) and SH protein sulfhydryl group in SH-SY5Y cells. (**A**) GSH; (**B**) GSSG; (**C**) GSH/GSSG ratio; (**D**) SH protein sulfhydryl group. The results are expressed as mean ± SEM from four independent experiments. * *p* < 0.05, ** *p* < 0.01, and *** *p* < 0.001 compared with control; ^§^ *p* < 0.05 and ^§§^ *p* < 0.01 compared with 6-OHDA.

Because of these effects and given the role of reduced glutathione (GSH) as one of the most important intracellular defenses against oxidative damage, a specific analysis of the effect of all treatments on intracellular GSH and oxidized glutathione (GSSG) levels was carried out. The results showed that CNF1 per se did not introduce any significant GSH perturbations within cells, whereas 6-OHDA exposure induced a 46% drop in GSH concentration compared to controls (Figure 4A). In addition, pre-treatment with CNF1 partially reverted the 6-OHDA effects (−36% vs controls) (Figure 4A). When GSSG was considered (Figure 4B), a 2.9-fold increase of this molecule was evident after 6-OHDA treatment. Also, in this case, CNF1 pre-treatment was partially able to prevent the increased levels of GSSG due to 6-OHDA exposure, with a 1.6-fold increase of this molecule with respect to controls. The overall perturbation of thiol balance is better represented in Figure 4C, in which the effects of all treatments are reported as the GSH/GSSG ratio. It can be observed that 6-OHDA treatments induced a strong and significant reduction of this value, as a result of the consistent soluble thiol oxidation process. CNF1 pre-treatment was able to at least partially restore this redox indicator. The results obtained on GSH homeostasis prompted us to investigate the influence of treatments on protein-bound sulfhydryl groups, the loss of which is considered a downstream event of GSH depletion. As shown in Figure 4D, a consistent and significant disappearance (−66% of controls) in protein thiols (SH) after the addition of 6-OHDA to the incubation medium, was detected. CNF1 pre-treatment was able to partially prevent this drop (−32% of controls). Overall, these results indicate that 6-OHDA-treated cells have been shifted into a pro-oxidant state, compared to untreated cells. This condition was partially restored if cells were pre-loaded with CNF1.

### 2.4. CNF1 Triggers Autophagy in SH-SY5Y Cells

**Figure 5:**
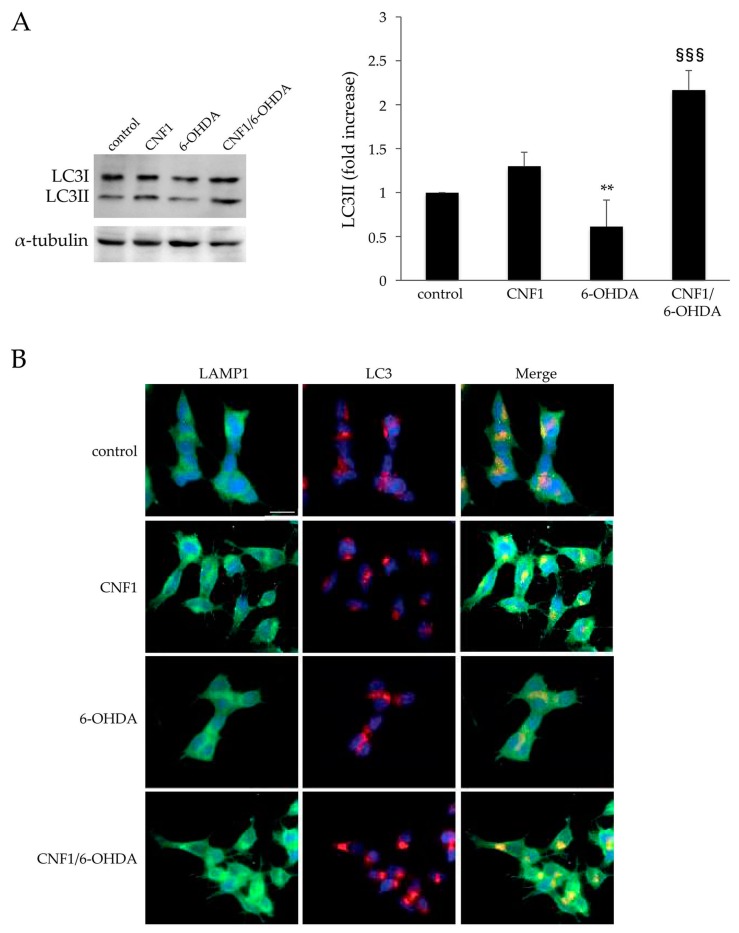
CNF1 triggers autophagy in SH-SY5Y cells. (**A**) Immunoblots (left panel) showing microtubule-associated proteins 1A/1B light chain 3 (LC3) (I and II) expression in SH-SY5Y cells. The amount of LC3II isoform is normalized as a function of α-tubulin (histogram, right panel). Note that 6-OHDA challenge reduced LC3-II levels and that CNF1 pre-treatment was able to counteract such a decrease. The graph reports the mean ± SEM from three different experiments. Results are expressed as fold increase relative to control (=1). ** *p* < 0.01 compared with control; ^§§§^ *p* < 0.001 compared with 6-OHDA. (**B**) Fluorescence microscopy images of SH-SY5Y cells co-stained with anti-lysosomal-associated membrane protein 1 (LAMP1) (green), anti-LC3 (red) antibodies and with the nuclear dye Hoechst 33258 (blue). Note that there is a significant co-localization of LAMP1 and LC3 (merge) after treatment with CNF1 and in cells pre-treated with CNF1 before 6-OHDA treatment. Bar = 10 µm.

Finally, we decided to investigate the effect of CNF1 on the autophagic process, whose signaling pathways are known to involve the Rho GTPases [39] and ROS [40]. For this purpose, the expression level of the autophagic marker microtubule-associated protein 1A/1B-light chain 3 (LC3)-II was analyzed by immunoblotting. We found that CNF1 induced overexpression of LC3-II (Figure 5A) in untreated cells. This increase was particularly evident when cells were pre-treated with CNF1 and then challenged with 6-OHDA. In particular, the graph in Figure 5A shows that 6-OHDA challenge reduced LC3-II levels and that CNF1 pre-treatment was able to counteract such a decrease and also to double up the autophagic protein with respect to control cells. To strengthen our finding, we next checked, by fluorescence microscopy, the occurrence of a co-localization of the well-known lysosomal-associated membrane protein 1 (LAMP1) and LC3. In fact, intensified video microscopy (IVM) analysis after triple staining with anti-LAMP1 antibodies (green), anti-LC3 antibodies (red) and Hoechst dye (blue) revealed a significant co-localization of LAMP1 and LC3 in perinuclear dot spots (Figure 5B) after treatment with CNF1. Moreover, in accordance with immunoblotting analyses, the co-localization appeared to be more evident when cells were pre-treated with CNF1 before the 6-OHDA challenge. 

## 3. Discussion

Rho GTPases are key regulatory proteins involved in a plethora of cellular functions [41]. In the brain, they can affect neuronal development and survival as well as neurodegeneration [42]. Previous in vivo studies on mouse models of different neuroinflammatory diseases characterized by brain mitochondria impairment, showed a beneficial effect of CNF1, a Rho GTPases’ modulator protein, on different pathological markers, including mitochondrial free radical overproduction and synaptic plasticity [20,22,28,29,30].

Reports on the role of CNF1 on cell-free radical production have so far been conflicting probably because depending on the cell type. In fact, although previous studies indicate CNF1 as a ROS-dependent transcription enhancer in human epithelial cells [43], other studies show a protective effect of this bacterial toxin against mitochondrial free radical overproduction in the brain of a mouse model of Rett syndrome [29], a neurodevelopment disorder with defects in brain mitochondrial complex II activity. This in vivo study is in line with increasing evidence that an impaired balance of mitochondrial fusion-fission [44,45,46,47,48,49] and the generation of ROS are involved in the pathology of neuroinflammatory diseases [50].

In this context, a better understanding of the processes allowing CNF1 to protect neuronal survival from oxidative stress may provide new strategies against neuroinflammation. To this end, we have investigated the possible preventive CNF1 effect on SH-SY5Y cells, a human neuroblastoma cell line, exposed to a strong oxidative stress by 6-OHDA treatment, the pro-oxidant derivate of dopamine that is widely used in ROS-induced dopaminergic cell death model system [31]. Data herein reported show that when SH-SY5Y cells are pre-treated with CNF1, their viability increases with respect to cells challenged with 6-OHDA alone and, also, confirm the toxin’s anti-oxidative effects [29] and the ability of CNF1 to elongate mitochondria [18,51] through Drp1 phosphorylation-dependent mitochondrial fission inhibition [18,20]. In fact, it is known that CNF1 can activate complex signaling that is mostly dependent on Rho GTPases activity modulation, such as the phosphoinositide 3-kinase (PI3K)/Akt/IκB kinase/NFκB pathway [51] and the cyclic adenosine monophosphate (cAMP)–PKA pathway [18]. Acting on PKA, CNF1 can phosphorylate Drp1 at Ser637, thus controlling the mitochondrial shape through impairment of mitochondrial fission [52,53]. Once fission is inhibited, the balance of mitochondrial dynamics is shifted towards unopposed mitochondrial fusion, resulting in a mitochondrial elongation and in an increase in ATP production [18]. The CNF1 ability to enhance mitochondrial functionality may also explain the moderate toxic effect that this bacterial protein appears to have on control SH-SY5Y cells (Figure 1B). In fact, as in other tumoral cell types, energetic metabolism of SH-SY5Y cells is altered compared with normal cells, resulting in a high aerobic glycolytic ’Warburg’ phenotype and dysregulation of mitochondrial activity [54]. Thus, the outcome of the CNF1-triggered mitochondrial fusion may be a system with mitochondrial dysfunction that may also provoke an increase in ROS generation, with consequent cell toxicity. On the other hand, the CNF1 effect on mitochondrial fusion could preserve cell viability against 6-OHDA, which induces a Drp1-dependent mitochondrial fragmentation in SH-SY5Y cells [32]. Hence, the partial CNF1 protective effect against 6-OHDA could be the result of CNF1 and 6-OHDA opposite activities on the same pathway. 

Since it has been hypothesized that Rho GTPases are involved in autophagic signaling pathways [39] and that ROS are key determinants in autophagy [40], we decided to investigate the effect of CNF1 on the autophagic process. In 6-OHDA-treated SH-SY5Y cells pre-incubated with CNF1, we have observed that ROS production is not increased, whereas auto-phagolysosomes production is favored. An essential factor for autophagosome formation is represented by the LC3 protein that exists in two different forms, the unlipidated cytosolic form (LC3-I), and the lipidated form (LC3-II) that localizes to autophagosomal membranes. Hence, the augmented expression of LC3-II observed in cells pre-exposed to CNF1 strongly supports the involvement of autophagy in the cell response to the toxin. Autophagy is a transcendental homeostatic process in which certain cell components are incorporated by double-membraned auto-phagosomes and degraded to produce energy or to preserve the cellular homeostasis and its viability. Autophagy breaks down damaged cellular components, such as non-functioning organelles and aggregated proteins, whose accumulation within cells can lead to deleterious effects [55]. Alterations in the autophagic cycle rate (flux), which begins with the formation of the phagophore and ends with the degradation of auto-phagosome cargo after its fusion with the lysosome, are commonly observed in response to stress [56]. In fact, in most cases, the induction of autophagy in response to stress acts as a pro-survival mechanism in neuronal cells [40,57,58,59], while very few examples exist where autophagy has been clearly demonstrated to mediate cell death [60,61]. This phenomenon could be caused either by induction of non-selective autophagy due to a pseudo-starvation response (stress) or to a stimulation of the autophagic degradation pathway due to the Drp1 phosphorylation, then involving mitochondrial dynamics. Both autophagic strategies do not involve mitophagy [62,63].

Despite both CNF1 and 6-OHDA treatments trigger an increase in ROS level, we hypothesize that the inhibition of mitochondrial fission, due to toxin pre-treatment, could induce macroautophagy activation, facilitating the neuroprotection against ROS [64]. The increased autophagy activity induced by CNF1 may preserve the redox homeostasis, thus influencing the cellular antioxidant response, as demonstrated by specific redox indicators such as both SOD/CAT and GSH/GSSG ratios, and protein SH groups content. The involvement of this kind of macroautophagy in the CNF1 ability to counteract oxidative stress and cell viability may also be confirmed by results shown in Figure 5, where the lysosome perinuclear position is increased by treatment with the toxin. Indeed, lysosomal positioning seems to play a pivotal role in coordinating autophagic flux [65]. Moreover, it is worth noting that the actin cytoskeleton, a principal target of CNF1, is crucially involved in several forms of intracellular trafficking by promoting the vesicular biogenesis and transport, while increasing evidence highlights the pivotal role of membrane–cytoskeleton interactions in macroautophagy [66].

In conclusion, the present study adds novel information about the mechanism of action of CNF1 on an in vitro model of PD, almost partially explaining some of the specific toxin effects on animal models of neurological diseases characterized by oxidative stress and mitochondrial dysfunction. Our results, which suggest a close link between CNF1 activity on mitochondria and on macroautophagy, and its antioxidant strategy, pave the way to the hypothesis that CNF1 pre-treatment, by triggering stress-related defensive cell strategies (i.e. autophagy activity and mitochondrial fusion), could “instruct” SH-SY5Y cells to counteract 6-OHDA toxic action. Further investigation, both in vivo or in vitro, will be necessary to prove the potential value of CNF1 in PD specifically and to deeper understand the molecular mechanisms involved in toxin activity.

## 4. Materials and Methods 

### 4.1. CNF1 Preparation

CNF1 was obtained from the 392 ISS strain (kindly provided by V. Falbo, Rome, Italy) and purified essentially as previously described [67] with few modifications in the procedure. Briefly, bacteria were grown overnight in LB medium, collected by centrifugation and resuspended in 50 mM sodium phosphate buffer (PBS) pH 7.4. Bacteria were sonicated and the homogenate centrifuged at 12,000 rpm for 30 min at 4 °C. The supernatant was precipitated with 50% ammonium sulfate. The precipitate was then extensively dialyzed against 25 mM Tris buffer pH 7.4 and subjected to three chromatography steps, using three different columns (Pharmacia, Piscataway, NJ, USA) equilibrated with the same buffer: (i) a gel filtration HiTrap Capto-DHEAE (5 × 5 mL) ion-exchange column; (ii) a gel filtration Superdex 75 xk16 column; (iii) a gel filtration Q Sepharose xk16/10 column. At the end of each column passage, eluted fractions were tested for CNF1 activity on cultured HEp-2 cells and also applied to an SDS polyacrylammide gel (8%), to verify the presence of CNF1 protein in each fraction. CNF1-containing fractions were then pooled and precipitated with 50% ammonium sulfate. The precipitate was dialyzed and applied to the next column. At the end of the procedure, purified CNF1 was subjected to SDS gel electrophoresis and quantified using BSA standards. CNF1 stock solution was then frozen in small aliquots at −80 °C in Tris-HCl 25 mM pH 7.4, containing 30% glycerol.

For treatments, CNF1 was diluted directly into the culture medium. For the first experiment, two doses of CNF1 were used, 1 × 10^−9^ M and 1 × 10^−10^ M. Since no differences were observed between the two concentrations, for the following experiments, only 1 × 10^−10^ M dose was used.

### 4.2. Cell Culture and Drug Treatment Procedures

SH-SY5Y cells (human dopaminergic neuroblastoma, ATCC® CRL-2266™, Manassas, VA, USA) were grown in Dulbecco’s Modified Eagle’s Medium supplemented with 10% fetal calf serum (FBS, Thermo Fisher Scientific, Waltham, MA, USA), 100 U mL^−1^ penicillin and 100 μg mL^−1^ streptomycin (Thermo Fisher Scientific, Waltham, MA, USA) at 37 °C in a 5% CO_2_ atmosphere, and treated with purified CNF1 toxin 2 hours before 6-OHDA addition (Sigma-Aldrich, St. Louis, MO, USA). For 6-OHDA treatments, the frozen power was first dissolved, just before the treatments, in saline solution (NaCl 0.9%) to obtain a 1000x stock solution and then diluted into the culture medium. We first tested two doses of 6-OHDA: 50 and 25 μM. We chose 25 μM 6-OHDA because 24 h of treatment with this concentration is non-lethal but does alter cell viability and morphology (Figure 1). 

### 4.3. Cell Viability

To evaluate the CNF1 effect on cell viability, we used the MTS assay system (Promega, Madison, WI, USA) according to the manufacturer’s protocol. Briefly, 10^4^ cells were seeded in a 96-well plate, and after 48 h, the medium was replaced with CNF1 10^−10^ M in 100 µL medium. After 2 h, 6-OHDA 25 µM was added to the selected wells. Twenty-four h later the medium was changed and 20 µL of MTS reagent was added to each well and left to incubate at 37 °C for 2 h. The absorbance at 490 nm was measured using a Tecan GENios plate reader (Tecan Group, Cernusco sul Naviglio, Milan, Italy). Control cells were cultured in the same way without CNF1 or 6-OHDA. Absorbance was expressed as a percentage of control. Wells without cells were used as blank and absorbance values were subtracted as background.

Cell viability was also determined by a trypan blue assay (Thermo Fisher Scientific, Waltham, MA, USA). Briefly, cells were detached from the culture dish with 10 mM EDTA (pH 7.4) and 0.25% trypsin in PBS (pH 7.4), were counted by using a typan blue (80 µM; Thermo Fisher Scientific, Waltham, MA, USA) exclusion method, based on the principle that live cells possess intact cell membranes that exclude certain dyes, including trypan blue. 

### 4.4. Immunofluorescence Analysis

Control and treated cells were fixed with 4% paraformaldehyde (Carlo Erba, Milan, Italia) and then permeabilized with 0.5% Triton X-100 (Sigma-Aldrich, St. Louis, MO, USA). After washings, cells were incubated with the following monoclonal antibodies (mAb) or polyclonal antibodies (pAb), alone or in combination, for 1 h at 37 °C: mAb anti-LAMP1 (Santa Cruz Biotechnology, Santa Cruz, CA, USA) and pAb anti-LC3 (MBL International, Woburn, MA, USA). After washings, cells were incubated with anti-mouse AlexaFluor 594-conjugated and anti-rabbit AlexaFluor 488-conjugated antibodies (all Thermo Fischer Scientific, Waltham, MA, USA) for additional 30 min at 37 °C. All samples were counterstained with Hoechst 33258 (Sigma-Aldrich, St. Louis, MO, USA) and mounted with Dako Fluorescent Mounting Medium (Dako, Santa Clara, CA, USA). The images were acquired by IVM with an Olympus fluorescence microscope (Olympus Corporation of the Americas, Center Valley, PA, USA), equipped with a Zeiss charge-coupled device camera (Carl Zeiss, Oberkochen, Germany). 

For mitochondrial staining, cells were incubated with 1 µM Mitotracker Red CMXRos (Invitrogen, Carlsbad, CA, USA) for 1 h before fixation and then processed for fluorescence microscopy.

### 4.5. Western Blot

Cells were lysed in boiled Sample Buffer 1X (50 mM Tris-HCl pH 6.8, 2% SDS, 10% glycerol, 100 mM DTT). Twenty-five or fifty μg (for pDrp1) of total protein extracts were resolved on 8%, 10% or 12% SDS-PAGE and electrically transferred onto polyvinylidene difluoride membranes (Bio-Rad Laboratories, Hercules, CA, USA). Membranes were blocked with TBS-T (20 mM Tris-HCl pH 7.4, 150 mM NaCl, 0.02% Tween-20) containing 5% skimmed milk (Bio-Rad Laboratories, Hercules, CA, USA), for 1 h at room temperature, and then incubated overnight at 4 °C with primary antibodies diluted in TBS-T containing 2% milk or 5% BSA. MAb anti-Drp1 (BD Biosciences, San Jose, CA, USA, 1:1000), pAb anti-pDrp1 (Cell Signaling Technology, Boston, MA, USA, 1:1000), mAb anti-OPA1 (BD Biosciences, San Jose, CA, USA, 1:500), pAb anti-Mfn2 (Cell Signaling Technology, Boston, MA, USA, 1:1000), pAb anti- LC3 (MBL International, Woburn, MA, USA, 1:1000) and mAb anti-α-tubulin (Sigma-Aldrich, St. Louis, MO, USA, 1:10,000) primary antibodies were used. After extensive washing in TBS-T, immunocomplexes were detected with Horse Radish Peroxidase conjugated species-specific secondary antibodies (Jackson Laboratory, Bar Harbor, ME, USA) followed by enhanced chemiluminescence reaction (Millipore Corporation, Billerica, MA, USA). Reactive bands were detected by the ChemiDoc MP system (Bio-Rad Laboratories, Hercules, CA, USA), and signal quantification was performed using the IMAGE LAB 5.0 software (Bio-Rad Laboratories, Hercules, CA, USA). Proteins were normalized as a function of α-tubulin. To quantify pDrp1, we first normalized total Drp1 content as a function of α-tubulin and, subsequently, we quantified pDrp1 signal using the normalized total Drp1 as an internal control.

### 4.6. Determination of SOD and CAT Activities

The enzymatic activity of SOD (E.C. 1.15.1.1) was determined by the quantification of pyrogallol auto-oxidation inhibition, as previously described [68]. The results were expressed as units per mg cell proteins. One unit of enzyme activity was defined as the amount of enzyme necessary for inhibiting the reaction by 50% at 25 °C. Auto oxidation of 0.2 mM pyrogallol in air-equilibrated 50 mM Tris-cacodylic acid buffer, pH 8.2, containing 1 mM diethylenetriaminepentaacetic acid was measured by an increase in absorbance at 420 nm. The enzymatic activity of CAT (E.C. 1.11.1.6) was assayed by Aebi’s method, whereby H_2_O_2_ decomposition to yield water and oxygen was measured at a wavelength of 240 nm [69]. The results were expressed as units per mg cell proteins. One unit of enzyme activity was defined as the amount of enzyme necessary to decompose 1 µmole of hydrogen peroxide to oxygen and water per minute at pH 7.0 at 25 °C at a substrate concentration of 10 mM H_2_O_2_.

### 4.7. Determination of GSSG, Reduced GSH Glutathione

To determine the total intracellular glutathione content, an enzymatic recycling assay with glutathione reductase (type IV, Sigma-Aldrich, St. Louis, MO, USA) and 5,5’-dithiobis-2-nitrobenzoic acid (DTNB, Sigma-Aldrich, St. Louis, MO, USA) was used [70]. For the measurement of GSSG, the acidified homogenates were submitted to derivatization with undiluted 2-vinylpyridine (Sigma-Aldrich, St. Louis, MO, USA) in the presence of triethanolamine (Sigma-Aldrich, St. Louis, MO, USA) for 1 h at room temperature. Samples were then assayed using the same procedure described above for total GSH measurement. The amount of reduced GSH present in the samples was calculated as the difference between total GSH and GSSG levels. The data were expressed as nmoles of GSH or GSSG per mg of cell protein.

### 4.8. Determination of Protein Sulfhydryl Groups

Protein sulfhydryl (SH) groups were evaluated as already described [71]. Cell pellets previously precipitated in ice-cold 5-sulfosalicylic acid were washed twice with 5% of the same solution and finally suspended in 0.5 M Tris-Hcl, pH 7.6. DTNB (100 μM final concentration) was then added and, after 20 min, the absorbance was measured at 420 nm. Data, expressed as nmoles of SH per mg of protein, were calculated on the basis of a GSH calibration curve.

### 4.9. Protein Quantification

Protein concentration was measured using the commercially available “Bio-Rad Protein DC Assay” (Bio-Rad Laboratories, Hercules, CA, USA), according to the manufacturer’s instructions. Bovine serum albumin was used as a standard.

### 4.10. Statistical Analysis 

Data are presented as mean ± SEM. Statistical analysis was performed by one-way ANOVA. When a significant interaction was detected, we also performed Tukey’s test for post-hoc analysis. *p* < 0.05 was considered as a threshold for a significant difference.

## Abbreviations

6-OHDA	6-hydroxydopamine
CAT	Catalase
CNF1	Cytotoxic necrotizing factor 1
Drp1	Dynamin-related protein 1
DTNB	5,5’-dithiobis-2-nitrobenzoic acid
GSH	Reduced glutathione
GSSG	Oxidized glutathione
IVM	Intensified video microscopy
LAMP1	Lysosomal-associated membrane protein 1
LC3	Microtubule-associated proteins 1A/1B light chain 3
mAb	Monoclonal antibodies
pAb	Polyclonal antibodies
PD	Parkinson’s disease
pDrp1	Phosphorylated dynamin related protein 1
PKA	Protein kinase A
ROS	Reactive oxygen species
SOD	Superoxide dismutase
